# A framework for enhancing pharmaceutical integrity and patient safety: novel mobile health solution integrating smart packaging and computer vision

**DOI:** 10.1038/s41598-026-38215-1

**Published:** 2026-02-18

**Authors:** Harshvardhan K, Vrisha Parikh, Prithviraj S K, Sanjay D, Rajay Vedaraj I S

**Affiliations:** 1 Department of Biotechnology, School of Bio Sciences and Technology, Vellore Institute of Technology, Vellore, India; 2 Department of Sensor and Biomedical Technology, School of Electronics Engineering, Vellore Institute of Technology, Vellore, India; 3 Department of Information Technology, School of Computer Science Engineering and Information Systems, Vellore Institute of Technology, Vellore, India; 4 Department of Mechanical Engineering, School of Mechanical Engineering, Vellore Institute of Technology, Vellore, India; 5 Department of Quantum AI, School of Computer Science and Engineering, Vellore Institute of Technology, Vellore, India

**Keywords:** Pharmaceutical authentication, Mobile Health (mHealth), Computer vision, Counterfeit drug detection, Supply chain integrity, Computational biology and bioinformatics, Health care, Mathematics and computing, Medical research

## Abstract

Counterfeit and substandard pharmaceuticals represent a critical global health crisis, with the World Health Organisation (WHO) reporting that falsified medicines comprise 10% of the global pharmaceutical trade, constituting one of the fastest-growing grey economies worldwide. This illicit market affects all regions, including high-income countries, causing devastating health and economic consequences that contribute to increased mortality and morbidity rates. The problem is particularly severe in developing nations with inadequate regulatory systems. Mobile health (mHealth) technologies have emerged as promising solutions for enhancing pharmaceutical supply chain integrity. However, comprehensive frameworks that integrate multiple authentication mechanisms remain limited in addressing the growing counterfeit drug crisis. The primary objective of this study is to design and implement a novel mobile health framework that integrates innovative packaging technology and computer vision to enhance pharmaceutical integrity and patient safety. This innovative approach enables consumers to authenticate genuine drugs and detect counterfeit/spurious products through advanced Quick Response (QR) code verification systems and artificial intelligence- powered tablet recognition capabilities. The proposed system utilises individual strip QR codes that enable real-time scanning at the point of sale to verify drug authenticity, directly addressing critical gaps identified in current pharmaceutical authentication methods. To overcome the practical challenge of expiration date loss when medication strips are cut at pharmacies, we developed a computer vision-based Artificial Intelligence (AI) model that automatically recognises the number of tablets remaining in a strip and correlates this information with the unique Identifier (ID). This approach leverages recent advances in computer vision for pharmaceutical applications and automated packaging inspection technologies. Each medication strip is assigned to an individual customer at the time of purchase, with pharmacists recording detailed customer information to ensure comprehensive tracking and accountability throughout the pharmaceutical supply chain. The integrated mobile application creates a robust anti-counterfeiting ecosystem by combining secure QR code authentication with intelligent visual recognition capabilities. The computer vision model provides accurate tablet counting and strip identification, maintaining continuity of medication tracking even when packaging is modified during dispensing processes, thus significantly enhancing supply chain transparency. This dual-authentication approach builds consumer confidence in pharmaceutical authenticity while directly addressing critical vulnerabilities identified in current regulatory frameworks. This comprehensive mobile health solution provides a scalable, evidence-based approach to pharmaceutical authentication that can be readily implemented across diverse healthcare systems globally, offering substantial potential for reducing the circulation of falsified medicines and improving patient safety outcomes in the ongoing battle against the pandemic of counterfeit pharmaceuticals. To overcome the practical challenge of expiration date loss when medication strips are cut at pharmacies, we developed a computer vision-based Artificial Intelligence (AI) model that automatically recognises the number of tablets remaining in a strip and correlates this information with the unique Identifier (ID). This approach leverages recent advances in computer vision for pharmaceutical applications and automated packaging inspection technologies. Each medication strip is assigned to an individual customer at the time of purchase, with pharmacists recording detailed customer information to ensure comprehensive tracking and accountability throughout the pharmaceutical supply chain. The integrated mobile application creates a robust anti-counterfeiting ecosystem by combining secure QR code authentication with intelligent visual recognition capabilities. The computer vision model provides accurate tablet counting and strip identification, maintaining continuity of medication tracking even when packaging is modified during dispensing processes, thus significantly enhancing supply chain transparency. This dual-authentication approach builds consumer confidence in pharmaceutical authenticity while directly addressing critical vulnerabilities identified in current regulatory frameworks. This comprehensive mobile health solution provides a scalable, evidence-based approach to pharmaceutical authentication that can be readily implemented across diverse healthcare systems globally, offering substantial potential for reducing the circulation of falsified medicines and improving patient safety outcomes in the ongoing battle against the pandemic of counterfeit pharmaceuticals.

## Introduction

Spurious drugs are those in circulation that appear to be the original but differ in composition and effectiveness^1–14^. They may have a wholly different or decreased quantity of the active ingredient. A false/fictitious company can manufacture these drugs under the pretence of the original manufacturer or even by seemingly legitimate manufacturers to cut costs and make greater profits. In India, there are strict guidelines to identify spurious drugs, mentioned under Section 17B and Section 33EEA of the Drugs Act. Most of the spurious drugs are manufactured in India, China and Russia, with considerable illegal manufacturing units being set up in the Philippines and Nigeria^15^. The identification of spurious drugs is quintessential due to the adverse effects they can have on consumers. Use of counterfeit medicines has severe consequences, including treatment failure and increased mortality and morbidity^16^. Not only will they slow the efficiency of treatment or not cure the disease they are meant to treat, but they can also cause long-term allergic reactions or effects in patients that can be fatal. According to a report published by the Ministry of Chemicals and Fertilisers, India’s pharmaceutical industry is considered the third-largest in the world by volume. According to a report, the European Commission has stated that 75% of global Spurious/Falsely-labelled/Falsified/Counterfeit drugs (SFFC) cases originate from India^17^. The Indian pharmacy market is also home to nearly 35% of these counterfeit medicines. The local markets of Uttar Pradesh, Bihar, Gujarat, and West Bengal are the most prone to the availability of spurious drugs^18^. Therefore, developing new methods for effectively identifying and removing these drugs is crucial. One such method is to make the consumer aware of the genuineness of the drug they are purchasing at the time of purchase through a transparent centralised system that cannot be altered or cheated by individual pharmacists. This paper focuses on one such solution, which uses innovative packaging, a scanning system-based mobile application, and a computer vision-based AI application. Each authorised manufacturing unit can access the application through a manufacturer’s login. When the manufacturer adds the details of the drug they are manufacturing and the number of strips (say n strips), many QR codes with unique IDs (n QR codes) will be made. These QRs will be printed on the drug strips by the manufacturer. At the time of purchase, the pharmacist scans the QR code that reveals the unique ID. This ID provides the basic details of the drug, such as the expiration date and drug composition, while also serving as verification that the specified drug is genuine. Any counterfeit or spurious drug will not reveal details upon scanning, as the application will not recognise a QR code it did not produce. QR scanning in each pharmacy will be performed using a system provided to all pharmacies. It will feature an ESP32 camera module to capture images of the sold strip and scan the QR code. Once the image is taken, it is pushed to the cloud to run through an Open Source Computer Vision (OpenCV) Model that recognises the number of tablets cut from the strip for sale. Identification of cut tablets is essential for keeping track of tablets from the strip. When the QR code is scanned, and the pharmacy collects the customer’s data, the unique ID of the strip is correlated with the customer’s information in the mobile application. The application then sends an Short Message Service (SMS) to the customer alerting them of the expiry of the purchased drug. Thus, this solution enables recognition of spurious drugs and efficient expiry tracking and alert systems.

## Survey analysis

**Fig. 1:**
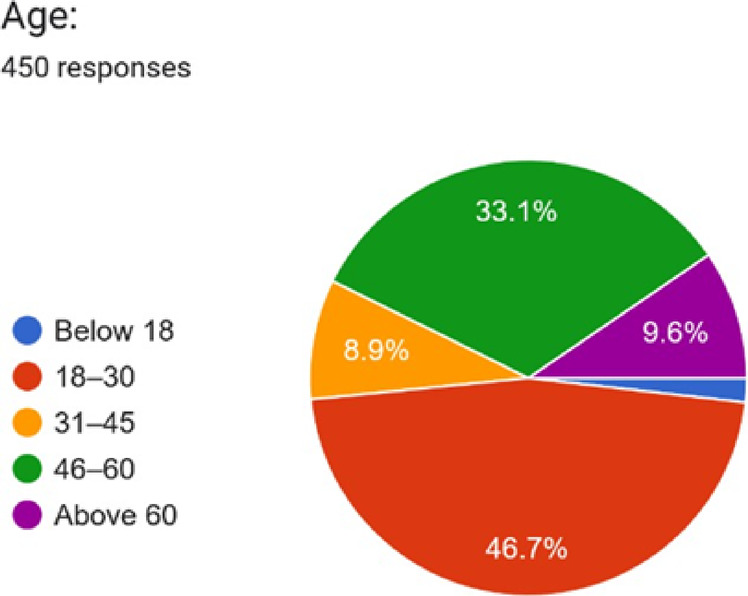
Percentage of Individuals in each age group across India.

**Fig. 2:**
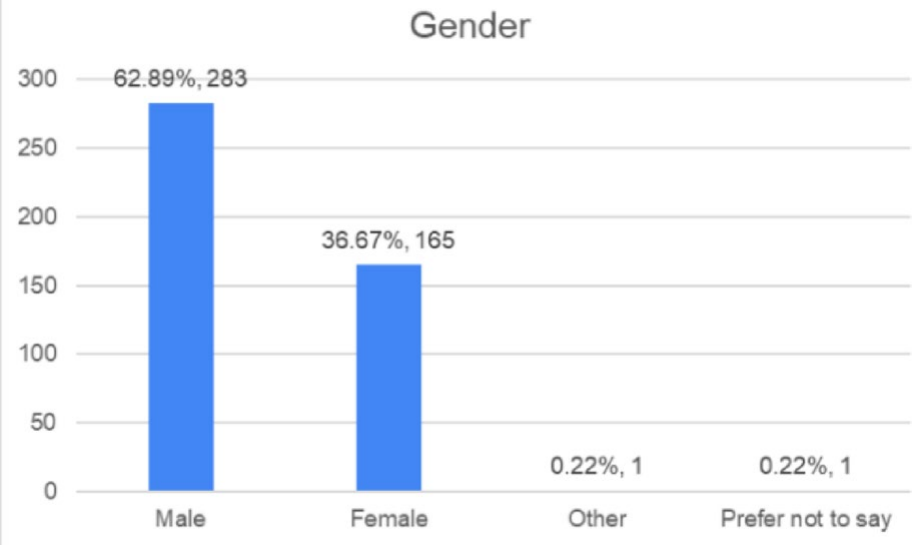
Gender of Respondent.

**Fig. 3:**
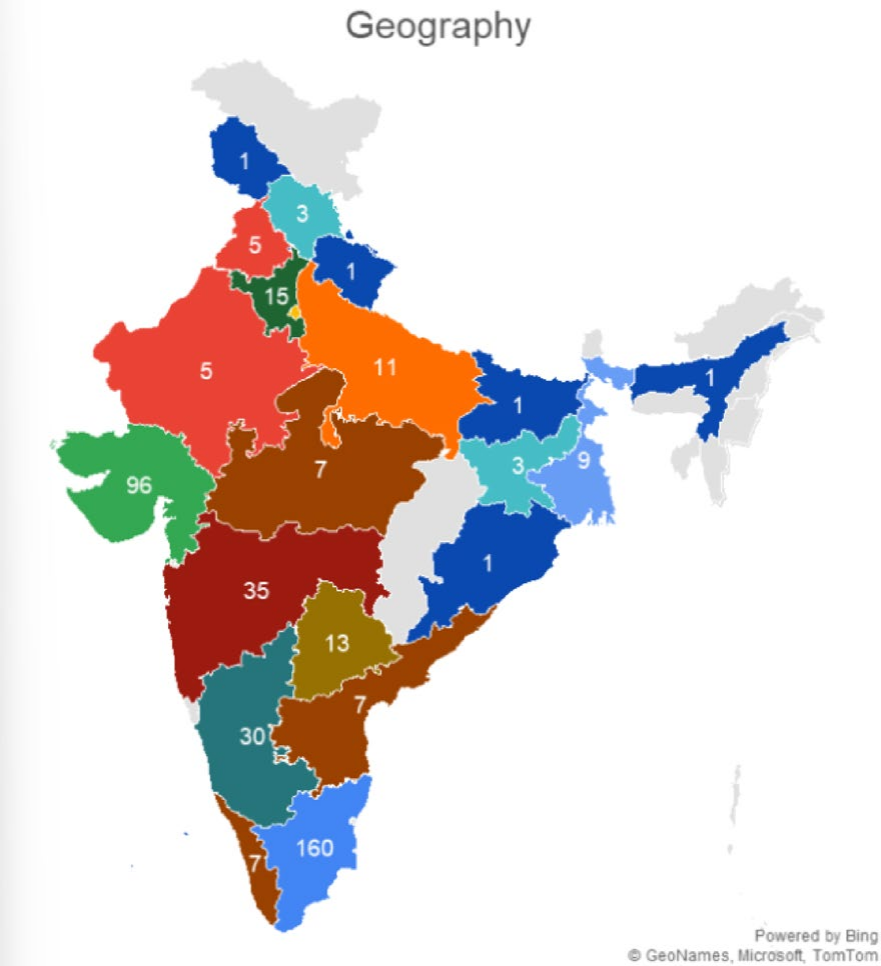
Number of submissions from each state. The map’s numbers show responses per state; colours have no significance.

**Fig. 4:**
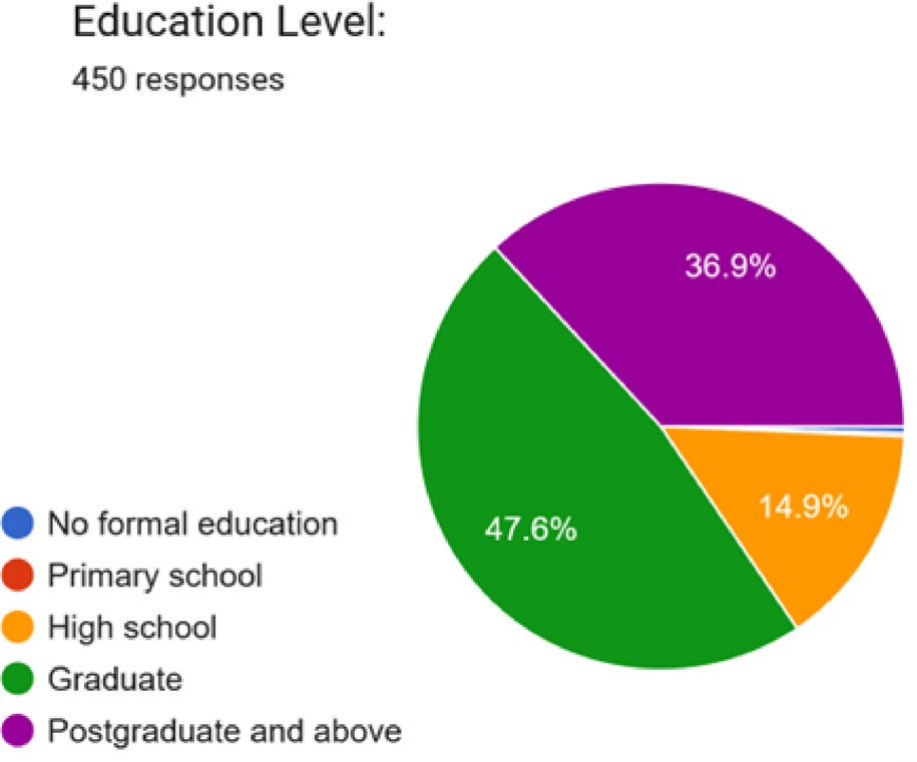
Percentage of individuals in each education level.

We circulated a comprehensive questionnaire to understand India’s current pharmaceutical consumption patterns, medication management practices, and consumer vulnerabilities to counterfeit medicines. From the 450 responses received, we mapped respondent demographics and their medication-related behaviours, establishing the empirical foundation for our integrated mobile health solution, which combines smart packaging, QR-based traceability, and computer vision technology. Table 1 presents the distribution of respondents across different age ranges. Table 2 outlines the gender-wise distribution, while Table 3 highlights the number of respondents from each state. Table 4 summarises the respondents’ highest level of education. Figures 1, 2 and 3, and 4 visually represent the information from the tables in charts for more straightforward interpretation.

**Table 1:** Age of Respondents.

Age Group	Frequency	Percentage
Below 18	8	1.78%
18–30	210	46.67%
31–45	40	8.89%
Above 60	43	9.56%
46–60	149	33.11%
Total	450	100%

**Table 2:** Gender of Respondents.

Gender	Frequency	Percentage
Male	283	62.89%
Female	165	36.67%
Other	1	0.22%
Prefer not to say	1	0.22%
Total	450	100%

**Table 3:** State of Residence.

State/Country	Frequency	Percentage
Tamil Nadu	160	35.56%
Gujarat	96	21.33%
Maharashtra	35	7.78%
Karnataka	30	6.67%
Delhi	23	5.11%
Haryana	15	3.33%
Telangana	13	2.89%
Uttar Pradesh	11	2.44%
West Bengal	9	2.00%
Other Indian States	34	7.56%
International (USA, Australia, Canada, Tanzania, Poland)	14	3.11%
Total	450	100%

**Table 4:** Education.

Education Level	Frequency	Percentage
High School	67	14.89%
Graduate	214	47.56%
Postgraduate and above	166	36.89%
No formal education	2	0.44%
Primary school	1	0.22%
Total	450	100%

The respondent pool is predominantly young (48.45% aged under 31, with the largest group being 18–30 years at 46.67%). The geographic concentration in Tamil Nadu (35.56%), Gujarat (21.33%), and Maharashtra (7.78%)—accounting for 64.7% of responses—reflects India’s urbanised pharmaceutical consumption centres. This demographic composition validates our application’s multilingual support strategy and simplified, accessible User Interface/User Experience (UI/UX) design, which caters to diverse digital literacy levels.

## Hypothesis framework: survey-driven feature implementation

Our survey findings directly informed the design and implementation of each feature within our mobile health platform. Rather than proposing theoretical solutions, each hypothesis and corresponding application feature emerged from empirical evidence of specific user vulnerabilities. The technical architecture and comprehensive implementation details of these features are elaborated in the section “Proposed solution”.

### Hypothesis 1

Unregulated Home Pharmacy—Digital Inventory Management and End-to-End Traceability.

The common practice of household medicine storage extends significantly beyond benign over-the-counter (OTC) products to include a high prevalence of potent, prescription-only medications (POMs), particularly antibiotics and medications for chronic illnesses. This behaviour transforms homes into unregulated, informal pharmacies, posing substantial public health risks related to improper self-medication, antimicrobial resistance (AMR), and consumption of expired or degraded medicines.

#### Survey evidence

97.8% of respondents confirm they keep medicines at home, establishing near-universal prevalence. High-risk prescription medications are widespread: 54.9% store chronic illness medications (Blood Pressure (BP), Diabetes) and 44.9% store antibiotics. Combined POM storage: 99.8% of respondents maintain at least one category of prescription-only medication. This demonstrates that the “home pharmacy” phenomenon is mainstream, not marginal.

#### Application features implemented

QR Code Registration & Traceability: Unique strip IDs with QR codes enable batch-to-consumer traceability from production to patient. Retailer Verification: QR scan validates legitimacy and expiry status; warns against expired medication before sale. Digital Inventory Dashboard: Cloud-based centralised record of all household medications with batch numbers, expiry dates, and purchase history. Secure Authentication: One Time Password (OTP) based authentication prevents medication mix-ups and unauthorised access; multi-user profiles are planned. Transaction History: Complete accountability from registration to delivery; enables rapid recalls.

### Hypothesis 2

Inadequate Consumer Verification—Real-Time Authenticity Verification via Backend Integration.

Consumer strategies for verifying medication authenticity rely overwhelmingly on inadequate proxies—such as vendor trust and superficial data checks (expiry dates, packaging appearance)—rather than robust product-level verification. This vulnerability is exacerbated by the prevalence of local pharmacies as the primary purchasing channel, making them susceptible to sophisticated counterfeits that can mimic standard packaging and expiration dates.

#### Survey evidence

72.2% purchase exclusively from local pharmacies, relying on a trust-dependent channel. 87.1% depend on non-verifying methods such as checking expiry or manufacturing dates (54.7%) or simply trusting the pharmacy (32.4%). Only 7.3% carefully check packaging quality, and just 2.4% use technological solutions like QR codes. Manufacturing dates and expiry information can be easily falsified on counterfeit packaging, rendering these methods unreliable for detecting chemical adulteration or subpotent active pharmaceutical ingredients.

#### Application features implemented

Point-of-Sale Verification QR scan triggers backend verification against the Supabase database, confirming the following: a legitimate manufacturer batch, no duplicate sales, the current expiry status, and active recalls/alerts. A digital pedigree creates a complete transaction record from manufacturer to consumer, forming an immutable chain; counterfeits cannot replicate verified Universally Unique Identifiers (UUIDs) in the database. Consumer Confidence Medication data delivered only after backend verification against manufacturer records—cannot be forged through packaging mimicry. Secure Application Programming Interface (API) & Middleware Dedicated middleware server verifies all database requests; strips ID (non-sensitive) and displays in QR code while protecting database access.

### Hypothesis 3

At-Home Medication Identification Failure—Persistent Digital Registry with AI Tablet Recognition.

The predominant method for at-home medication identification—visual inspection of the drug strip—is fundamentally unreliable. Nearly total reliance on this single method is undermined by two compounding failures: (1) significant legibility challenges due to small print, and (2) near-total loss of identifying information when tablets are separated from the original strip, a standard household practice.

#### Survey evidence

93.1% rely almost exclusively on “looking at the name on the strip”—a single point of failure. 31.8% report that the text on medicine strips is “Difficult” or “Very difficult” to read. 78.9% have found it challenging to identify individual tablets once removed from the original strip. This creates a high risk for accidental misidentification, taking the wrong medicine, or making medication errors during self-administration.

#### Application features implemented

Digital Capture at Point-of-Sale QR scan captures and stores structured metadata (drug name, dosage, composition, expiry, and batch number), tablet images, and identifying characteristics (colour, shape, and imprint codes) via cloud synchronisation. Persistent Cloud Lookup Complete medication record accessible anytime through app—independent of packaging condition, legibility, or tablet separation. No reliance on physical packaging.

### Hypothesis 4

Expiry Vigilance Paradox—Expiry Monitoring and Timely Alerts.

Self-reported diligence in checking medication expiry dates is an unreliable predictor of actual safe use. A critical intention-behaviour gap exists: while 75.1% claim to “always” check expiry dates, 43.4% have either consumed or are unsure if they have consumed an expired medicine. Manual checking, despite stated vigilance, is insufficient due to cognitive burden, legibility challenges, and information loss after packaging separation.

#### Survey evidence

75.1% of respondents claim to “Always” check expiry dates, while 43.4% have either consumed or are unsure about consuming expired medication. The gap between these figures (43.4% vs. 24.9% inconsistent checkers) reveals that mistakes still happen even among vigilant users. The reasons for failure include the cognitive burden of keeping track of dozens of medications, poor legibility, and the loss of information after separation.

#### Application features implemented

Automatic expiry capture and cloud indexing involve expiry dates being automatically recorded through QR/barcode scanning or manufacturer data linked to strip IDs; these are securely stored and synchronised across devices via Supabase PostgreSQL. The visual urgency dashboard provides a colour-coded medication overview: green indicates safe, yellow signifies expiring in more than 30 days, and red shows expired or expiring within 7 days, allowing quick visual assessment from the home screen. The expiry alert system ensures that time-based notification alerts are never missed, taking into account the expiry of medications.

### Hypothesis 5

Market Opportunity—Unified, Integrated Mobile Health Platform.

A significant market opportunity exists for a dedicated, comprehensive medication management application. The vast majority of users currently employ no digital tools, and those who do rely on inadequate, non-specialised solutions (generic note-taking apps, calendars). Simultaneously, users express high demand for a purpose-built application with integrated, robust features.

#### Survey evidence

87.8% use no application to track medicines, while 12.2% rely on generic tools such as note-taking apps, calendars, or repurposed e-pharmacy apps—none of which are designed for post-purchase medication management. 77.1% affirm they would find it helpful to have a dedicated all-in-one application. Meanwhile, 94.6% express comfort with camera-based scanner technology, with 77.3% being comfortable and 17.3% somewhat comfortable. Users also clearly demand an integrated feature set, including detailed tablet notes, tablet identification, authenticity checking, and expiry notifications.

#### Application features implemented

Comprehensive Feature Integration: Single application unifying: Consumer interface (inventory, QR scanning, expiry monitoring, purchase history, notifications), Retailer interface (stock management, QR scanning, customer transactions, tablet verification), Manufacturer interface (QR registration, batch management, recall broadcasting). 2. Cross-Platform Accessibility: Flutter/Dart development enables seamless deployment on iPhone Operating System (iOS) and Android, a consistent user experience, and a single codebase, thereby reducing maintenance overhead. 3. Multi-Language Support: Flutter localisation ensures accessibility across India’s linguistic diversity. 4. Simplified Onboarding: OTP-based mobile authentication via Supabase with minimal UI; immediate core feature access post-authentication. 5. Unified Dashboard & Notifications: Home dashboard summarising expiry alerts, inventory status, and purchase history; customizable notifications by product category and user preferences.

### Hypothesis 6

Patient Vulnerability Through Information Deficit—Integrated Safety Features and Access Controls.

Patient vulnerability to adverse medication events is significantly amplified by dual information deficits: (1) widespread failure to check for legitimate side effects, and (2) inability to verify drug authenticity. This forces patients to rely solely on the trustworthiness of the supply chain, leaving them unable to assess or react to unexpected symptoms that could stem from known side effects, counterfeit medicines, or medication errors.

#### Survey evidence

42.7% either do not check for side effects (36.9%) or do not know where to find this information (5.8%). 26.9% of all respondents reported experiencing unexpected symptoms after taking medicine. 19.8% checked for side effects and noticed symptoms; 7.1% noticed symptoms without checking. Qualitative responses reveal: acknowledged ignorance about counterfeit detection, substitution of trust for verification, and blurred causal attribution (inability to distinguish counterfeit effects from legitimate side effects or misdiagnosis).

#### Application features implemented

Manufacturer Notifications include manufacturers broadcasting critical product information, recalls, safety alerts, adverse event warnings, and usage education, prominently displayed on the home screen for immediate visibility. Age & Quantity Restrictions enforce manufacturer-imposed limits on high-risk and controlled medications, preventing unauthorised transactions at the point of registration through eligibility verification.

## Case study

As noted above, the consumption of spurious drugs can be fatal. However, the scale of these effects varies significantly with the extent of falsification, the type of medicine falsified, and the longevity of consumption. It has also been challenging to quantify these effects due to incomplete or no reporting of such cases and the fact that the impact of taking a spurious drug ranges from fatal to almost no effects^19^. The exact effects depend on the consumption period, the extent of falsification, the target organ, and other factors. Here, we examine a case from Lahore, Pakistan, where a mass outbreak of pyrimethamine toxicity among patients with ischemic heart disease, resulting from industrial-level contamination of isosorbide mononitrate tablets, is seen. The batch of contaminated tablets contained approximately 50 mg of pyrimethamine per tablet. 664 cases of pyrimethamine toxicity were reported in cardiac patients (ages ranging from 58 to 75 years) who had recently received free drugs from the Punjab Institute of Cardiology, Lahore, Pakistan. Among them, 151 (22.7%) patients were reported dead, of which 116 (76.8%) were male. On examination of the tablets given to the patients, it was found that a batch of isosorbide mononitrate tablets was contaminated with 50 mg pyrimethamine^20^. Pyrimethamine, a folic acid antagonist, exhibits antimicrobial action against the causative agent of malaria and possesses sporontocidal action. It is also effective against the causative agent of toxoplasmosis. It is used for preventing malaria and treating toxoplasmosis^21^. The safe dose for pyrimethamine given to malaria patients is 25 mg/week, while the patients taking the spurious drug twice a day consumed 100 mg/day, which is 28 times more every week. Table 5 lists the causes of death as highlighted by autopsy reports of 3 patients out of the 151 reported to have died by consuming the intoxicated medications.

**Table 5:** Summary of autopsy findings in three fatal cases following pyrimethamine toxicity.

Case	Age	Gender	Autopsy Finding
1	58	Male	Acute coronary syndrome and ventricular fibrillation due to ischemic heart disease; drug-induced bone marrow suppression from pyrimethamine toxicity.
2	75	Male	Cardiopulmonary arrest due to advanced ischemic heart disease; major contributing factor: drug-induced bone marrow suppression.
3	65	Male	Cardiopulmonary arrest due to ischemic heart disease, aggravated by drug-induced bone marrow suppression (pyrimethamine toxicity).

Postmortem analysis of deceased patients’ samples confirmed the presence of pyrimethamine at toxic concentrations in the blood and urine. Symptoms presented by patients upon admission to hospitals included vomiting, diarrhoea, fever, abdominal pain, cough, pruritus, severe bone marrow suppression, neutropenia, skin hyperpigmentation, gum bleeding, haemorrhages, and oral ulcers. In this outbreak, fatal cases exhibited severe cytopenia, elevated bilirubin, coagulopathy, and mucosal and bone marrow pathology typical of exposure to toxic pyrimethamines^20^.

Studies such as these highlight the adverse effects spurious drugs can have on such a large part of the population. This highlights why they pose a danger to society and the necessity of answering the root cause.

## Background

Regarding absolute volume, India has been the largest consumer of antibiotics globally. However, the consumption of medications is not constant across the country; it is highly variable and affected by the diversity in population, age, structure, health system organisations, etc., across all states^22^. The consumption pattern has also changed overall each year for India. To understand the depth of how variable these trends are, we shall discuss these trends across 2011–2019. Table 6 summarises the consumption patterns across the time period at the national level. Relative change, Compound Annual Growth Rate (CAGR), and the median consumption are summarised in Table 6. DID refers to the Defined Daily Dose (DDD) of 1000 people per day.

**Table 6:** Summary of antibiotic consumption at the National level during 2011–2019.

Measures	Median Interquartile Range [IQR]	Relative Change (%)	CAGR (%)
DID Absolute	10.7 [10.6–10.9]	−3.6	−0.5
Access, %	25.9 [25.9–26.2]	−13.1	−1.7
Watch, %	51.8[49.6–53.1]	3.9	0.5
Reserve, %	0.4[0.4–0.8]	246.9	16.8
Discouraged, %	19.5[18.0–20.5.0.5]	11.3	1.3
Not Classified, %	0.9[0.9–5.2]	−52.0	−8.8
Fixed-Dose Combination (FDC), %	36.4[34.5–37.4]	0.9	0.1
National List of Essential Medicines (NLEM) listed, %	43.8[42.8–45.9]	3.2	0.4
Central Drugs Standard Control Organisation (CDSCO) approved, %	49.2[48.4–49.7]	6.2	0.8

Reproduced from^22^.

Methods for tracking drug expiration have been a development topic for many years. There have been attempts to regulate spurious drugs and to track expiry dates efficiently. However, implementing these solutions is not well addressed, especially in a vastly populated country like India, which has a diverse distribution of resources.

Existing solutions have been based on applications that are pharmacy-centric. They work like an inventory management system^23^ for the pharmacy and notify the pharmacists of the expiry of specific medicine batches. Additionally, they sometimes alert the consumer who bought that medicine through a notification^24^ by accessing the consumer database. However, most of them exist in theory, and using these services is the pharmacist’s initiative. Moreover, these systems are not a common sight in India. No single, central system can be implemented for the drug chain in India or elsewhere. Only individual applications are made by individual pharmacies specifically for their use only.

A significant problem with these individualistic platforms made by pharmacies is feeding the inventory details into the application. It is incredibly time-consuming for a pharmacist to feed individual batch expiry dates for each drug in the system. Secondly, there is no uniformity in data entry. As all the platforms are custom-made by individual companies/pharmacies, the data entered varies in format and specifications. Thus, it can not recognise and control spurious drugs in practical situations beyond serving as an inventory management system. Therefore, there is a need for a centralised system not just for inventory management but also to ensure that no spurious or counterfeit medicine is sold to a customer. Our solution aims to be a centralised system that is implementable in pharmacies nationwide.

In India, there exist two major problems that are overlooked: no uniformity in the supply chain of drugs in various regions and strip cutting. First, the supply chain management structure is highly decentralised and fragmented, with multiple distribution channels, leading to huge inefficiencies in the procurement, storage and distribution of medicines^25^. The number of intermediate steps after which a pharmacy gets a strip of medicine varies greatly depending on the size of the pharmacy, location, and supply to the area. Because of this, it isn’t easy to put a checkpoint at each intermediate transit point. This makes solutions based on tracking of the chain nearly impossible to implement. Second is strip cutting, in which the pharmacist cuts a few tablets from the strip to sell, a practice very commonly seen in countries like India. This poses a serious issue as tracking when selling strip fractions becomes increasingly complex. This issue won’t be addressed unless the pharmacist individually enters this detail. Additionally, this increases the chances of swindling, as this would be the perfect opportunity to swap out genuine medicine with spurious ones. Our solution considers these two major problems and addresses them to ensure the solution’s efficiency in handling spurious drugs.

There have been notable advancements aimed to improve certain aspects of the supply chain. The article^13^ proposed a pill intake detection tool that uses digital image processing to analyse images of a blister to detect the presence of pills. It shows how such a tool to recognise pills can be integrated into mobile health solution apps without needing additional hardware to track the actual numbers of pills consumed to improve medication adherence. While effective for it’s intended purpose, it was more reliable only for round shaped pills and needed improvement to accommodate various pill structure. Moreover, it is designed ed to monitor medication intake at an individual level rather than integration within the broader supply chain. Our focus, on the contrary, is to develop techniques that are integrable in the overall supply chain, accounting for the non-uniformity in the infrastructure. By leveraging deep learning based pill detection, our prototype enables pill counting to address the challenges posed by strip cutting.

Another study by^11^ proposed techniques for defect detection in pharmaceutical blister packages. It explains that conventional defect detection methods include human intervention to check the quality of tablets within the blister packages, which is inefficient, time-consuming, and increases labor costs. To mitigate this issue, the You Only Look Once (YOLO) family is primarily used in many industries for real-time defect detection in continuous production^11^. While it is a thorough approach for real time quality check of blister packaging, it is limited to controlled industrial settings and do not extend post sales verification or consumer level authentication. Our framework targets detection of fake medication at the point of sales, enabling post distribution verification through the integration of computer vision and secure QR based identification mechanism.

Our solution focuses on the supply chain’s start and end points, ensuring it works the same with any number of intermediate transfers. The manufacturer prints a QR code, and the pharmacy does the scanning. Thus, even if a spurious drug enters the circulation because of intermediate swindling, it will be immediately identified at purchase. The integrated scanning mechanism that takes the image of the strip while scanning the QR at the time of sale uses a You Only Look Once Version 5 (Yolo V5) based computer vision model that identifies the number of tablets left on the strip after cutting. The pharmacist in this case doesn’t have to manually enter this anywhere, ensuring there are zero possibilities of a scam. Thus, this proposed solution is designed to be implemented in a country like India and is intended to address all prevalent issues.

## Proposed solution

The existence of spurious drugs within the pharmaceutical supply chain poses a significant threat to public health and safety. By implementing and managing a secure universal database with unique identification of each medication unit, we address the issue by maintaining accountability at all times.

When a manufacturer produces a new batch of medicine, they can register their strip on our website, specifying details such as the name of the drug, expiry date, and other manufacturer information. This UUID ensures that every strip can be traced back to its origin. Thus, the system can establish end-to-end traceability by maintaining information on every transaction between a retailer and a customer. Introducing counterfeit drugs into the supply chain makes it significantly challenging, as every strip must correspond to a legitimate UUID in the database. The manufacturer can choose to receive the data to be included in the QR, which is linked with the unique ID, which they can use to design and print their own QR codes, or they can utilise our web app to create a template with which the app generates printable files. The web app’s simple and user-friendly interface allows quick and easy designing.

When a customer buys a strip, the retailer can process the transaction in one of two ways: either by scanning the QR code using their mobile device or by utilising the proposed Computer-Aided Design (CAD) model (detailed in the section "CAD model for enhancing mobile-assisted operational process"), which specifically addresses common reliability and accuracy issues associated with mobile camera scanning.

The UUID associated is verified with the backend systems. The app will also warn the retailer if the medicine has already expired. The retailer then enters the customer’s mobile number to send the corresponding data to their mobile phone. Customers can also be wary of counterfeit drugs and confirm the authenticity of the medicine they purchase, as they can receive the data only if the medicine is legitimate.

**Fig. 5:**
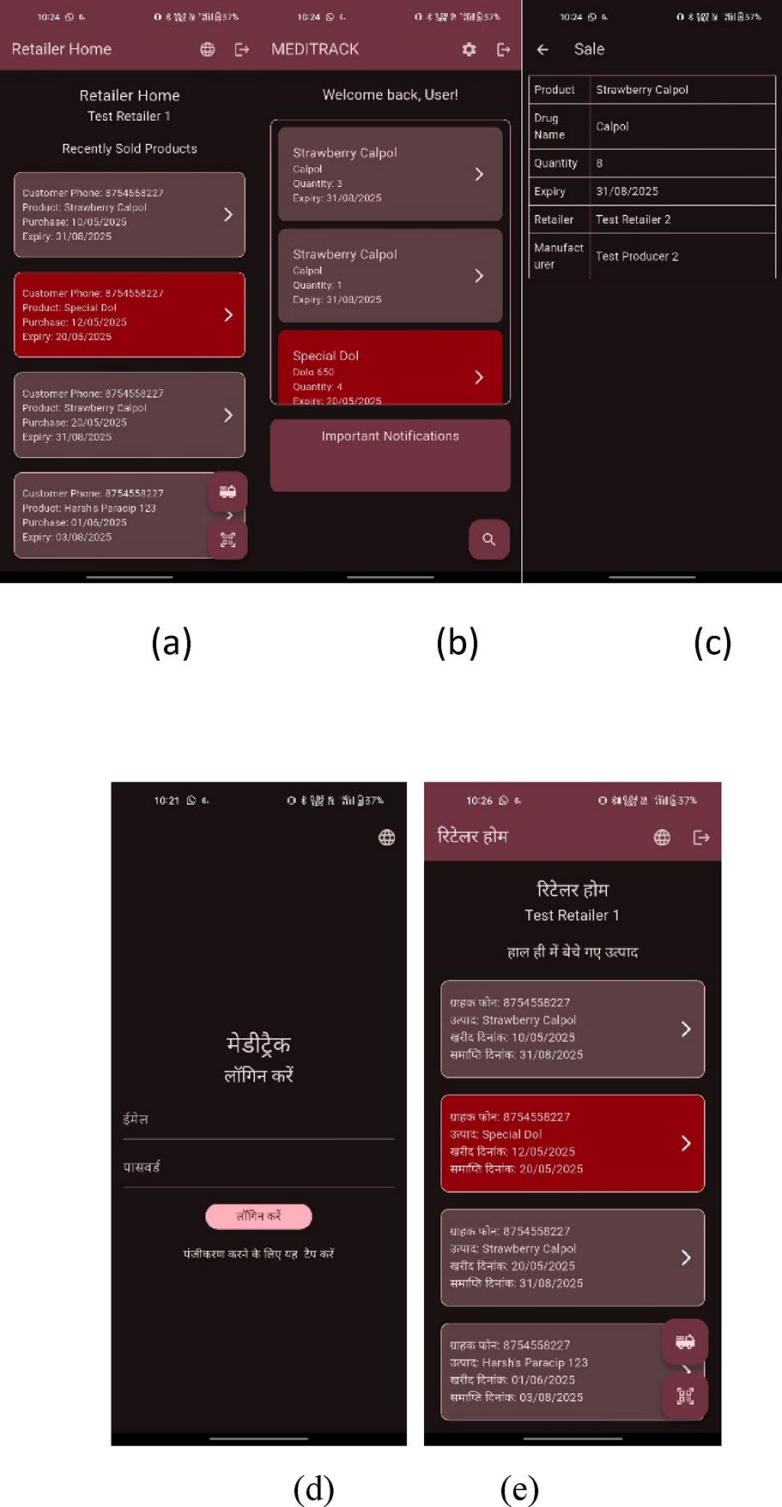
Prototype screenshots of proposed mobile app, featuring a multi-lingual user interface. **a** *Retailer Home Screen*—Primary home screen for retailers, showcasing a dashboard of recent sales, highlighting alerts and buttons to process new sales. **b** *Consumer Home Screen*—Landing page for consumers, featuring a dashboard of recent purchases with alert highlights in red, and a section for important global notifications. **c** *Consumer Sale Screen*—A view of a specific purchase made by the consumer, displaying the expiry date, quantity, and source details. **d** *Multi-Language Support*—Login screen rendered in Hindi, demonstrating multi-language localisation support in the system. Figure**e**: *Retailer dashboard in regional language*—A render of the retailer home screen in Hindi.

Expiry dates printed in strips also tend to get lost due to damage. This leads to the possibility of the consumption of expired drugs, resulting in complications. By implementing a simple mobile application to manage and store information, customers do not have to worry about checking expiration dates. The mobile application will provide timely alerts to warn users of expiring drugs and features a minimal user interface, as shown in Fig. 5.

### Functional and non-functional requirements

Basic functional requirements that are crucial for any mobile application deployed on a large scale include a clear and intuitive User Interface and User Experience, by implementing simple navigation throughout the app, displaying information clearly and concisely, ensuring responsive, accessible and aesthetic UI elements and user-friendly interactions such as swipes and gestures^26^.

All data transactions must be secure, efficient, and sensitive. Data is only accessed through the middleware server, which verifies the authenticity of all requests to the server. The mobile application stores non-classified data as cache on the device, which contains no sensitive user data. All data is securely stored in Supabase’s PostgreSQL database. PostgreSQL offers consistency and availability, following the CAP theorem. This ensures data is synchronised across regions and is available at all times.

Security is ensured by implementing user authentication with mobile number and OTP. Supabase offers simple and strong phone login authentication that allows us to easily implement mobile numbers with OTP login functionality in our app.

Core functionalities of the consumer app include the registration process, where consumers use their mobile number and an OTP is sent to their number to complete the registration. They can immediately start using the app features. Authentication is made secure by leveraging the features of Supabase’s authentication services.

Consumers can then view their purchase history and a summary of expiry alerts (Following the colour codes as mentioned in the section "Application features implemented") on the home dashboard. The consumer app also includes quick technical or medical support in the form of documentation.

The app supports multiple languages throughout the user interface, enabling maximum accessibility for users across India (as shown in Fig. 5 (d) and (e)). The app automatically translates to supported languages using a combination of localisation in Flutter and Google Translate API services.

Retailers also have their own mobile app, which requires a separate login. They can view a summary of current inventory and expiry alerts on the dashboard. They can update their inventory by scanning new arrivals using the QR scanning functionality. If a retailer scans the QR without installing the app prior, they will be redirected to the Play Store/App Store page of the retailer’s app. They can proceed with sales by utilising the app’s AI features or CAD model, ensuring accountability at all times. Retailers can then input the customer’s mobile number to forward the details to their device.

All QR codes contain ‘strip-id’, a unique identifier used to refer to the specific strip details in the database. The database can only be accessed via secure requests through our dedicated web server; thus, the strip-id is non-sensitive information^27^. We tested the end-to-end operation of the app using a low-level imitation of a printed QR code on a strip.

In addition to verifying drug authenticity, mobile applications also play a crucial role in preventing substance misuse, especially among minors, by enforcing restrictions on age or purchase quantity imposed by manufacturers on the registration of the product. As highlighted in this paper^28^, early access to controlled substances is a significant risk of substance abuse, especially among children. This safeguard ensures that high-risk medications are sold only if authorised by an adult. The proposed model by^29^ achieved an accuracy of 91.5% in recognising prescription details using a combination of OCR and machine learning classifiers^29^. As part of future work, we plan to integrate this detailed solution to allow verification of prescribing physician’s consent.

### Frameworks and tools used

The app utilises Flutter to facilitate cross-platform development and implement clean architecture in the Dart programming language. Flutter also allows easy development of fluid and user-friendly interfaces^30^. Our backend server is powered by the JavaScript framework Deno, which includes features such as native type safety using TypeScript, a faster runtime compared to frameworks like Node.js, and more.

## Features of the application

Digital applications have evolved to encompass various functionalities to streamline diverse user workflows. Yet, the proliferation of single-purpose tools has led to fragmented experiences and inefficiencies in task management. To address this challenge, our app portrays a feature-rich platform that integrates productivity, organisational, analytical, and collaborative modules within a unified interface, thereby reducing the need for multiple disparate applications. Unlike conventional solutions focusing on isolated functionalities, our application couples comprehensive scheduling with timely reminders and various other features that enable us to provide a cohesive end-to-end user experience. It enhances user efficiency by minimising context switching, supports scalability, and ensures adaptability.

### Secure authentication

To authorise users, we use OTP based authentication along with a validity check powered by Supabase. This helps prevent unauthorised access to the database and protects against possible misuse of the proposed AI solution. The app features a minimal user interface for both registration and login processes.

### Expiry date visibility

An accurate expiration date display informs users of when a medication retains its approved potency, purity, and quality under specified storage conditions. Presenting this information prominently reduces the risk of administering degraded or ineffective drugs and aligns with Food and Drug Administration (FDA) guidance on expiry-date labelling for consumer safety.

### End-to-end traceability (backtracking to consumer)

By capturing unique identifiers at manufacture and recording each handoff, the system builds a complete “digital pedigree” from production line to patient, enabling retrospective batch-level investigations if quality concerns arise. Such full-chain visibility enables recall management and counterfeit interception before the product reaches clinicians or end-users.

### Timely alerts and notifications

In addition to expiration alerts, the mobile application also shows important information published by manufacturers. These notifications can be customised for targeted audiences by applying filters based on individual products, categories of products for specific use cases, and other relevant criteria. The user interface is structured with core design principles to ensure users can quickly identify expired purchases and view important notifications directly from the home screen.

An early-stage prototype of the mobile application was developed to demonstrate and understand the core functionalities of the app. The application connects to a middleware over Representational State Transfer Application Programming Interfaces (RESTful APIs) to access the database and the Computer Vision (CV) model. Basic unit testing was performed with 15 distinct test cases along with the printed QR-code strip imitation, including but not limited to invalid email addresses, incorrect passwords, erroneous quantity values at sale, and incorrect phone numbers.

## Dataset

In this study, we utilised the Ultralytics Medical Pills Dataset^31^, a curated, open-source dataset designed for training object detection models in pharmaceutical applications. The dataset is particularly suited for tasks involving pill identification and counting, offering quality annotations, a consistent format, and diversity in pill arrangements and appearances. It was selected for its compatibility with the YOLOv5^32^ and You Only Look Once Version 8 (YOLOv8)^33^ frameworks and their focus on real-world pill layout scenarios.

### Dataset composition

The original dataset comprises 115 high-resolution Red, Green, and Blue (RGB) images containing pills in various configurations, including blister strips, tablet arrangements, and free-form pill groupings typically encountered in clinical and retail pharmaceutical environments. It follows the standard Ultralytics split, consisting of 92 training images and 23 validation images, and does not include an independent test set. To enable performance evaluation, we added 20 additional test images, which were manually annotated with ground-truth labels using LabelStudio. With these additions, the final dataset consists of 135 images in total. The detailed dataset composition is shown in Table 7.

**Table 7:** Properties of the Dataset.

Dataset Property	Value
Total Images	135
Training Set	92 images
Validation Set	23 images
Test Set	20 images
Image Resolution	Resized to 640 × 640
Class Labels	Single class – pill
Annotated Objects	1368 instances

### Annotation format

All images are annotated in YOLO format, where each annotation file corresponds to a single image and contains bounding box coordinates normalised between 0 and 1. A single object class — “pill” — is used for labelling. The annotations were created using manual annotation tools and are compatible with the YOLOv5/YOLOv8 training pipelines.

### Preprocessing and data augmentation

To improve the model’s robustness and reduce overfitting, YOLOv8’s built-in training pipeline applied several real-time data augmentation techniques. Mosaic augmentation combined four different images into a single frame, increasing spatial variety and enriching contextual information. Random horizontal flips helped the model learn from mirrored views, making it less sensitive to orientation changes. Scaling and cropping were used to adjust object sizes and aspect ratios, helping the network adapt to variations in scale. Brightness and contrast were slightly altered to expose the model to changing lighting conditions. Finally, Hue, Saturation, Value (HSV) jittering introduced small shifts in colour, enabling the network to handle natural variations in hue and saturation across real-world scenes.

All images were resized to 640 × 640 to match the model’s input size. These augmentations allowed the model to generalise better across different pill layouts and environmental conditions. However, we acknowledge that augmentation alone cannot fully compensate for the small size of the original dataset, and additional independent data would further strengthen generalisation.

### Challenges addressed by the dataset

The dataset addressed key challenges critical to real-world pill counting systems, including overlapping pills, partial occlusions, colour and shape variation, diverse lighting conditions, and reflections from blister packs. These variations help simulate practical imaging scenarios commonly encountered in pharmaceutical workflows. While the dataset captures important real-world challenges, its limited size means additional data would further strengthen robustness and real-world reliability.

These factors make the dataset useful for developing AI systems in pharmaceutical logistics, inventory tracking, and packaging automation.

## Machine learning approaches: supervised and unsupervised learning methods

Artificial Intelligence (AI) is increasingly vital in the pharmaceutical domain, particularly when analysing large, complex datasets. Within AI, machine learning methods are generally divided into two main categories: supervised and unsupervised learning, each offering different strengths depending on the problem and the nature of the data.

Supervised learning involves training models on labelled datasets, where the input-output relationships are clearly defined. This approach is beneficial for tasks such as pill identification, drug classification, and disease prediction, where annotated examples guide the learning process^34,35^. For instance, Convolutional Neural Networks (CNNs) have been used to classify pills by analysing features such as shape, colour, and imprint^36^. In contrast, unsupervised learning works without labelled data to uncover hidden patterns or natural groupings within the dataset. It finds applications in pharmaceutical research for clustering molecular structures, detecting anomalies in supply chains, and segmenting patients based on treatment response^36^. These techniques are instrumental in exploration analysis, especially when manual labelling is impractical or expensive.

In this study, we adopt a supervised learning approach, using the Ultralytics Medical Pills dataset^31^, which provides high-quality, annotated pill images. This enables the training of deep learning models to detect and classify pharmaceutical pills based on visual features. However, as the dataset is relatively small, the supervised model’s performance should be interpreted with consideration of potential generalisation limitations.

## Results and discussion

### Performance matrices

The performance of the YOLOv8 model was evaluated on the validation set after 100 epochs. The results demonstrate substantial precision and recall, indicating the model’s ability to detect and localise pills in images.

**Table 8:** Metrics of the YOLOv8 model.

Metric	Value
Precision	0.7978
Recall	0.7263
Mean Average Precision (mAP@0.5)	0.7672
mAP@0.5:0.95	0.4857

The values shown in Table 8 are based on the model’s first evaluation using the expanded dataset, which includes our independently annotated 20-image test set. These metrics reflect the model’s current performance on a relatively small dataset and therefore may not fully represent how the model will perform in real-world conditions.

### Confusion matrix

**Fig. 6:**
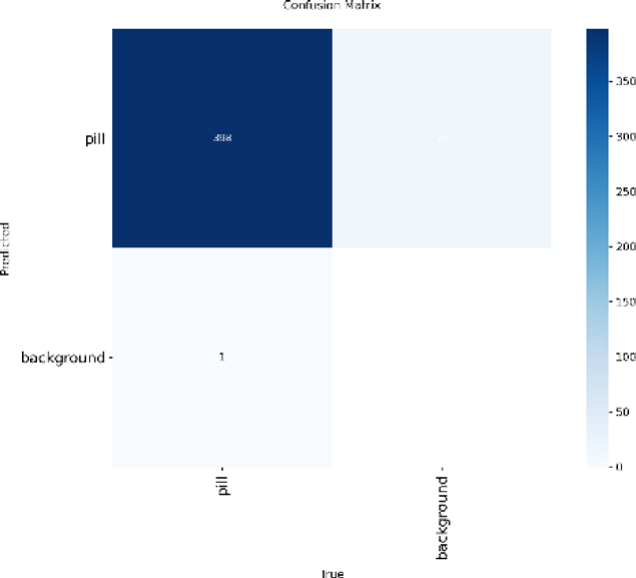
Confusion Matrix showing correct and incorrect predictions for the “pill” class.

The confusion matrix in Fig. 6 shows how effectively the model separates pill instances from the background. A high number of true positives (correctly detected pills) and true negatives (correctly identified background) indicates that the model is focusing on the right visual features and keeping misclassifications to a minimum. The very small counts in the false positive and false negative regions further suggest that the model rarely confuses pills with background elements. Overall, the results point to a model that detects pills with few false alarms or missed cases, which is important in applications where accurate counting is essential, such as medical and pharmaceutical settings. At the same time, these outcomes are based only on the available validation set, so evaluating the model on larger, independent datasets would help confirm how well it performs in real-world conditions.

### Precision-recall curve

**Fig. 7:**
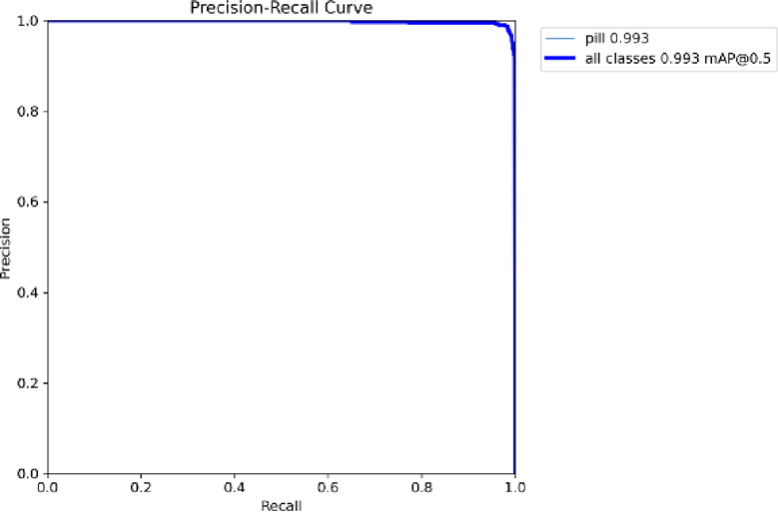
Precision-Recall curve indicating model stability across different confidence levels.

The Precision-Recall (PR) curve for the pill class is presented in Fig. 7. It shows consistent high precision across varying confidence thresholds.

### Loss and mAP over epochs

**Fig. 8:**
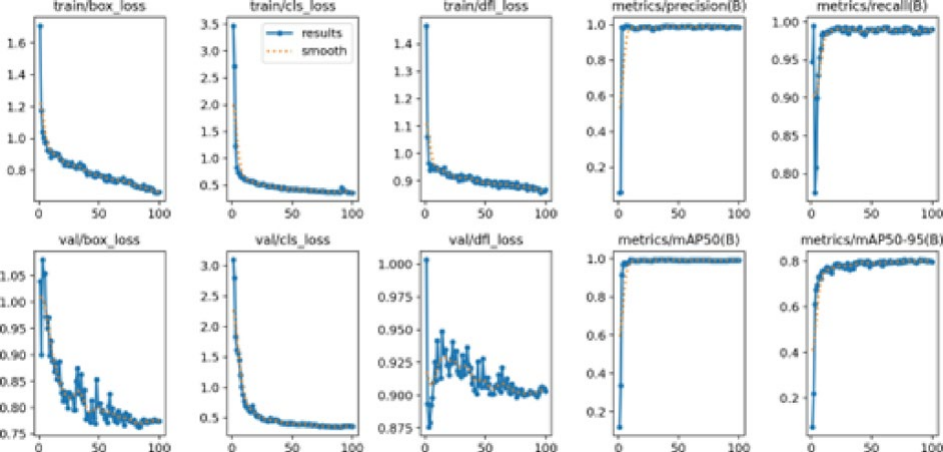
Model’s training loss and mAP. Over 50 epochs.

The model’s training loss and mAP over 50 epochs indicate stable convergence and improvement in accuracy, as shown in Fig. 8.

### Visualisation of prediction

**Fig. 9:**
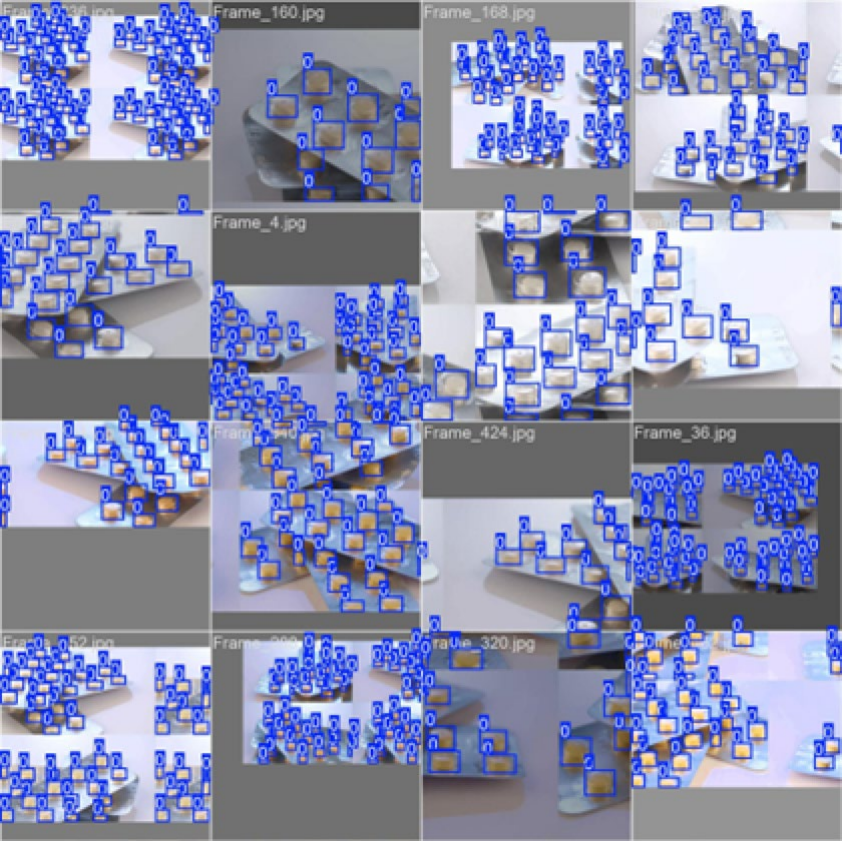
Predictions on a Training Batch.

**Fig. 10:**
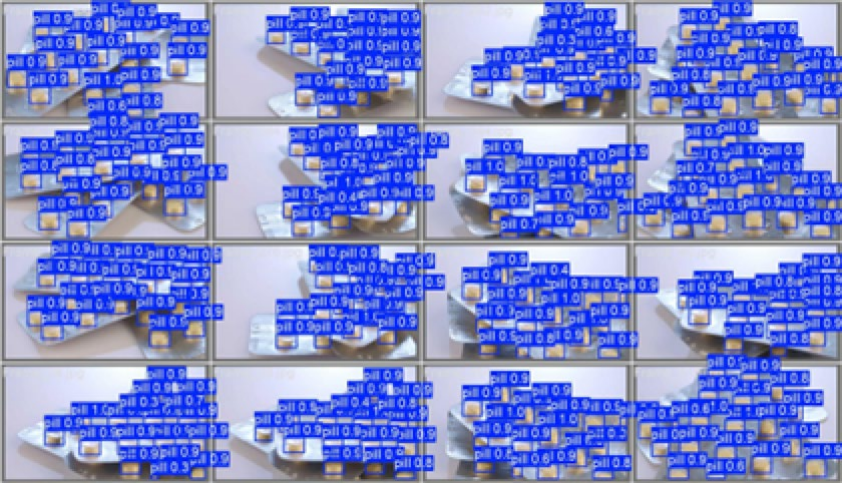
Predictions on a Validation batch.

We examined the model’s predictions on both the training and validation batches to get a clearer, more intuitive sense of how well it detects pills in different situations. As shown in Figs. 9 and 10, the model is able to pick out and localise pills across a range of scenes—different orientations, lighting changes, partial occlusions, and even visually busy backgrounds. The predicted bounding boxes match the ground-truth labels quite closely, which suggests that the model handles these variations reasonably well within the scope of the dataset.

**Fig. 11:**
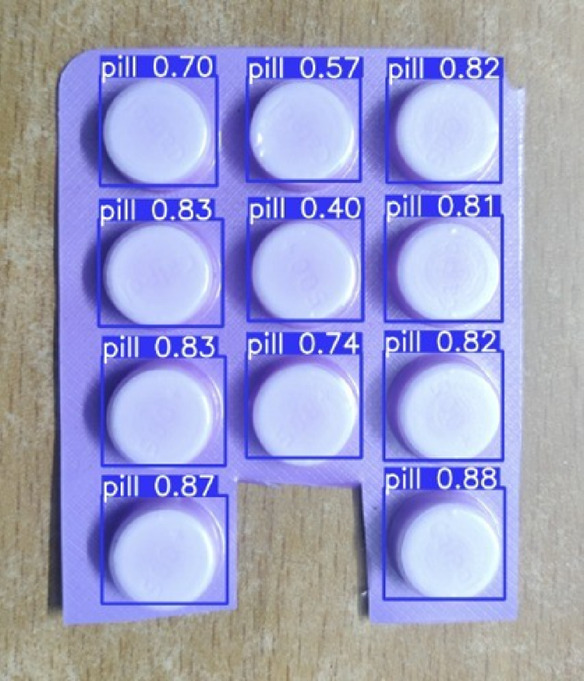
Real-time example of pill detection of Calpol 500 mg using our trained YOLOv8 model.

We also tried running the model in a small real-time setup to see how it might behave in an actual application. The example in Fig. 11 comes from the mobile interface, where the model detects and counts pills in a blister strip directly on the device. The count is displayed on the image, and the entire process finishes in under a second. This quick response hints at the model’s suitability for on-device use.

The combined results accurate localisation and consistent counting indicate that the system has good potential for settings like pharmaceutical packaging or inventory tracking. That said, these observations come from controlled tests and a limited dataset, so broader evaluation would still be helpful to understand how well it generalises in real-world use.

## CAD model for enhancing mobile-assisted operational process

### Objective of the CAD model

To make the mobile-based application more dependable during use, we designed a simple CAD model that keeps the tablet strip steady and helps the camera stay aligned. The idea was to create a small physical setup that guides the user holding the strip in place, capturing the image, sending it to the server, and giving a signal once the process is done. At this stage, the CAD model works mainly as a practical prototype to show how the system could function. Detailed measurements such as alignment accuracy or how the device behaves thermally, are planned for later stages of development.

### Tools

The choice of tools for this part of the project was driven by the need to design, test, and integrate the hardware in a straightforward way. SolidWorks was used to model the tablet holder and enclosure^37^, giving us good control over dimensions and how different components fit together. We also ran basic simulations using SolidWorks’ built-in analysis features to check for stress distribution, deformation, and overall stability under normal use. These simulations were mainly aimed at verifying that the concept is mechanically sound, while more in-depth, quantitative testing will be carried out as part of the next steps.

### Model description

The CAD model comprises two primary components: the main body and the top plate. The top plate is designed to house the electronic system, including sensors and imaging components. Careful consideration was given to material selection, ensuring the structure’s durability, lightweight characteristics, and ease of fabrication.

**Fig. 12:**
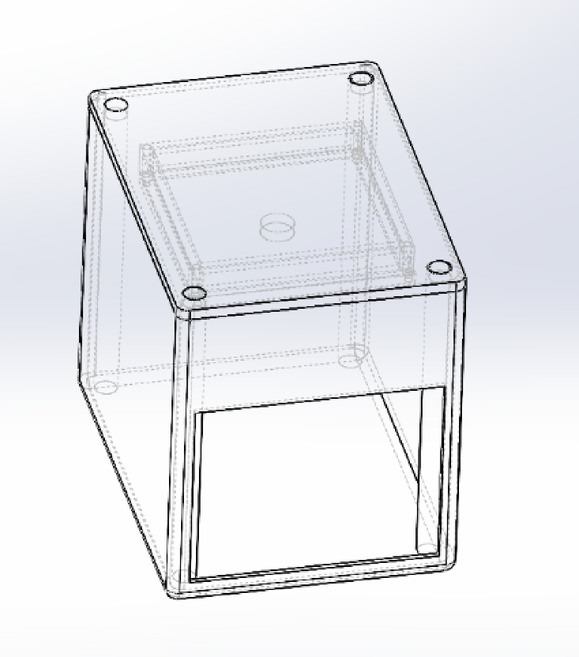
Isometric view of the model.

Mounts for sensors and other electronic components were strategically positioned to ensure optimal functionality and secure integration, as shown in the hidden lines in Fig. 12. The electronics layout was topologically optimised to maximise image acquisition quality, thereby improving the effectiveness of downstream post-processing tasks.

**Fig. 13:**
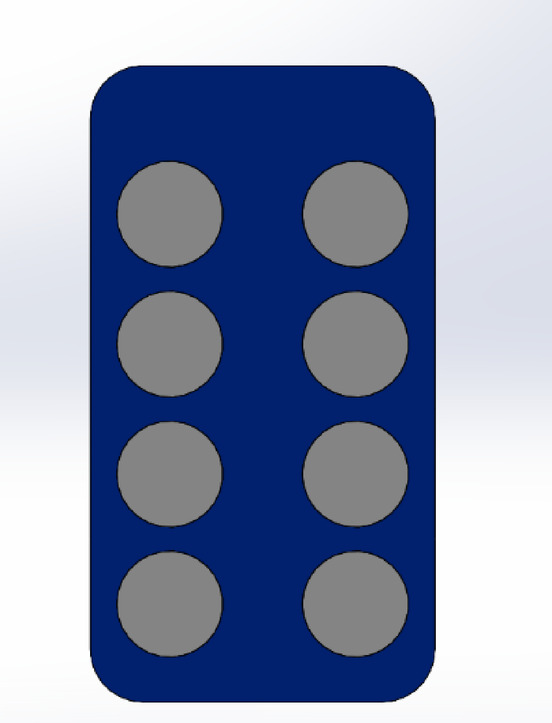
Model of tablet strip used.

From an ergonomic standpoint, the model was designed to allow easy insertion and alignment of strips or samples, ensuring consistent user interaction and minimising handling errors. The supporting structures were designed to provide mechanical stability while maintaining accessibility to critical components during operation and maintenance. A test tablet strip is modelled for testing, shown in Fig. 13.

The mechanical enclosure for the ESP32-CAM (Camera-Enabled Microcontroller) module and pill strip was designed using SolidWorks to ensure optimal alignment of the camera with the strip placement area. Key features include Material Selection: PLA (Polylactic Acid) was selected for 3D printing due to its ease of fabrication, dimensional stability, and eco-friendliness. Camera Mount: The camera slot was designed at a fixed focal distance to ensure consistent image clarity of pill strips. Strip Slot: A guided tray was designed to allow users to insert pill strips manually under the camera field of view. Ventilation Holes: Passive ventilation slots were added to prevent overheating of the ESP32-CAM during extended use. Port Access: Universal Serial Bus (USB) port and General Purpose Input/Output (GPIO) header cutouts were provided for programming and peripheral integration.

### Electronics architecture

To enable automated pill counting using a compact and low-cost system, an embedded vision setup was developed using the ESP32-CAM microcontroller. The following components were selected for the system: Microcontroller: ESP32-CAM (integrated Wireless Fidelity (Wi-Fi) + Bluetooth with onboard OV2640 camera; ideal for real-time image acquisition and lightweight AI tasks). Camera Module: OV2640 (2 megapixel (MP), embedded in ESP32-CAM; used for capturing images of pill strips). Power Supply: 5 V regulated supply (via USB or external adapter). Alert System: Piezo buzzer and LED module connected via GPIO to indicate missing or extra. Communication: Wi-Fi-enabled transmission of detection results to a mobile or web-based interface.

**Fig. 14:**
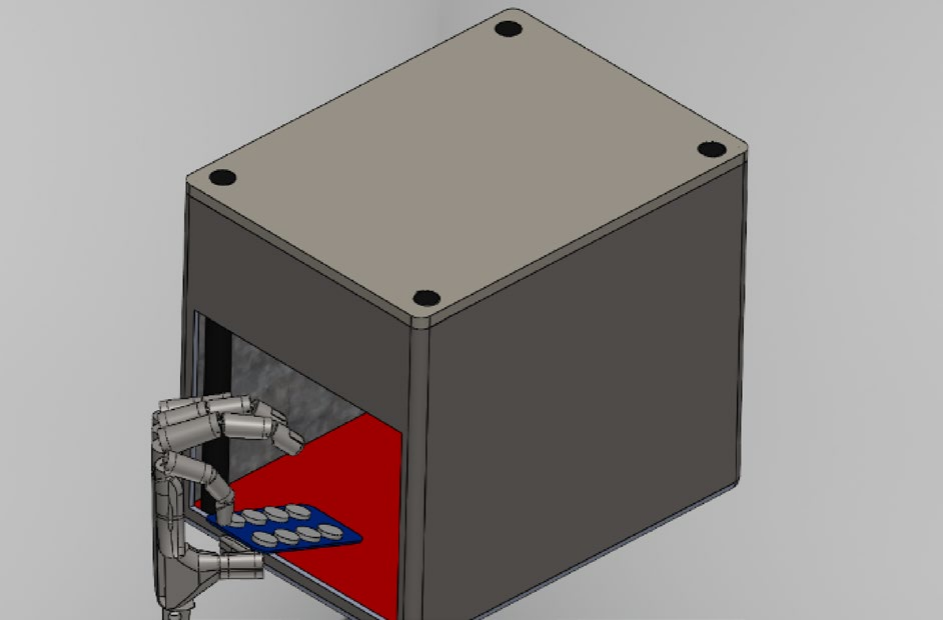
CAD Representation of the Proposed Design^35^.

**Fig. 15:**
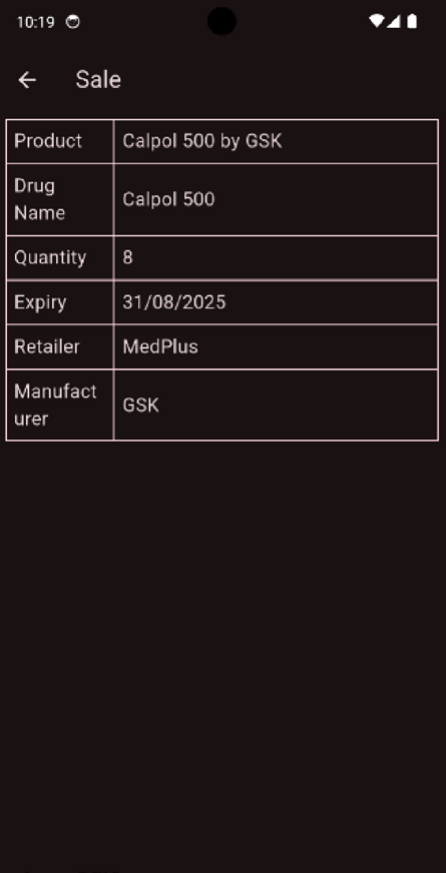
Mobile App Output for the Sample.

The ESP32-CAM served as the image acquisition unit and the processing interface with the trained YOLOv8 model running on an external system (or optionally cloud-connected for inference). The electronics were compactly housed within a custom-designed 3D-printed enclosure shown in Fig. 14. The Test output of the app is shown in Fig. 15.

Since this hardware setup is an early functional prototype, parameters such as latency, frame-capture timing, and long-duration thermal behaviour have not yet been fully characterised and will be addressed in future evaluations.

## Future prospects

The proposed solution lays a solid framework for removing spurious drugs from circulation in the markets and addressing the various challenges surrounding it. The next phase of work should focus on structured enhancements aimed at strengthening the reliability, scalability, and real-world integration of the current prototype.

The next primary step involves controlled deployment in selected pharmacies to evaluate the system’s performance under original conditions. This will allow assessment of scanning accuracy and pharmacy usability. Data collected from these deployments will guide the iterative refinement of the prototype’s structure and workflow, providing insights into real-time problems encountered.

Further work will also concentrate on improving model robustness by expanding the dataset to include a wider variety of tablet shapes, colours and packaging designs. This will improve the computer vision model and reduce failure rates in real-world implementation.

Finally, collaboration with regulatory authorities and stakeholders shall be explored to align the framework as a verification mechanism within the broader drug monitoring systems.

## Data Availability

The dataset generated and analysed during the current study is available in the dataset_project repository, https://github.com/sanjay2422-dot/dataset_project.The dataset analysed during the current study is available in the Ultralytics repository, https://github.com/ultralytics/ultralytics/blob/main/ultralytics/cfg/datasets/medical-pills.yaml.
